# MHC and KIR Polymorphisms in Rhesus Macaque SIV Infection

**DOI:** 10.3389/fimmu.2015.00540

**Published:** 2015-10-21

**Authors:** Lutz Walter, Aftab A. Ansari

**Affiliations:** ^1^Primate Genetics Laboratory, German Primate Center, Leibniz Institute for Primate Research, Göttingen, Germany; ^2^Department of Pathology and Laboratory Medicine, Emory University School of Medicine, Atlanta, GA, USA

**Keywords:** KIR, MHC class I, SIV infection, genetic association, rhesus macaque

## Abstract

Natural killer lymphocytes are essentially involved as the first line of defense against agents such as viruses and malignant cells. The activity of these cells is regulated via interaction of specific and diverse killer cell immunoglobulin-like receptors (KIR) with the highly polymorphic cognate MHC class I proteins on target cells. Genetic variability of both KIR and MHC-I ligands has been shown to be associated with resistance to many diseases, including infection with the immunodeficiency virus. Disease course and progression to AIDS after infection with human immunodeficiency virus-1 (HIV-1) is essentially influenced by the presence of the stimulatory KIR3DS1 receptor in combination with HLA-Bw4. Knowledge of such genetic interactions that contribute to not only disease resistance but also susceptibility are just as important. Such combined genetic factors were recently reported in the rhesus macaque AIDS model. Here, we review the rhesus macaque *MHC class I* and *KIR* gene systems and the role of their polymorphisms in the SIV infection model.

## Introduction

Natural killer (NK) cells are lymphoid cells that are part of the innate immune system. They are known to play important roles that include the elimination of infected cells (1), immune surveillance of cancer cells (2), the regulation of adaptive immune responses (3), and human reproduction (4). The activation of NK cells is regulated by input from both inhibitory and stimulatory receptors with many of those showing clonal expression patterns (5). NK cell receptors belong to two divergent protein families, the killer cell lectin-like receptors (KLR) and the killer cell immunoglobulin-like receptors (KIR), which are characterized by their extracellular C-type lectin-like and immunoglobulin-like domains, respectively. While keeping their KLR system relatively conserved (6), platyrrhine (New World monkeys) and catarrhine primates (Old Word monkeys, apes, human) have substantially diversified their KIR system (7–9). Important ligands for many KLRs and KIRs are members of the family of major histocompatibility complex (MHC) class I proteins. Both the KIRs and their natural cognate ligands display enormous genetic variability (10–12) and combinations of respective allotypes show differential binding strengths and can, therefore, substantially impact NK cell function (13–18). Consequently, certain polymorphisms of MHC class I ligands and KIR are associated with various diseases, including infections with the immunodeficiency viruses [for reviews, see Ref. (19, 20)]. Progression to AIDS is significantly delayed in HIV-1 infected individuals carrying the HLA-Bw4 serological epitope, in particular those having isoleucine at amino acid position 80 (Bw4-80I), and certain allotypes of the inhibitory KIR3DL1 or the activating KIR3DS1 receptors (20–22). These genetic and epidemiologic data were supported by *in vitro* functional data showing enhanced cytolytic activity of KIR3DS1+ NK cells toward HIV-infected Bw4-80I+ target cells and elevated control of HIV-1 replication compared to KIR3DS1− NK cells (23). The expansion of KIR3DL1+ and KIR3DS1+ NK cells that were dependent on the presence of the cognate Bw4 epitope were also monitored in patients during acute HIV-1 infection (24). Evidence for successful interaction between KIR3DS1 and HLA-Bw4-80I has only recently been documented (25) despite numerous attempts in the past. Interestingly, this interaction is peptide dependent and includes peptides from HIV-1 proteins pol and nef (25).

### *MHC Class I* Gene Polymorphisms and the Outcome of Experimental SIV Infection in Rhesus Macaques

The first human AIDS-like symptoms were observed in a colony of macaques (26). Subsequently, it was demonstrated that AIDS could be induced by experimental inoculation of naïve macaques with tissues from the diseased animals (27, 28). Since then infection of macaques with the simian immunodeficiency virus (SIV) has become an established and excellent animal model for studying HIV infection and AIDS. Similar to human HIV infection, macaques are not natural hosts of SIV and have therefore not adapted to it. Of the currently known 23 species of macaques, the rhesus macaques (*Macaca mulatta*), long-tailed (or cynomolgus) macaques (*Macaca fascicularis*), and pig-tailed macaques (*Macaca nemestrina*) are the predominant species utilized for studies following experimental infection with a number of SIV isolates (29, 30). Notably, rhesus macaques of Chinese and Indian geographic origin differ genetically (31) and these differences are expected to underlie the known phenotypic alterations of Chinese and Indian SIV-infected rhesus macaques [reviewed in Ref. (32)]. Particularly influencing disease course and progression to AIDS are polymorphisms of *MHC class I* genes due to their crucial role in regulating cytotoxic T cell and NK cell responses. Indeed, there are striking differences in the frequencies of *MHC class I* alleles between rhesus macaques of different geographic origin (33–35).

The *class I* genes of the rhesus macaque *MHC* (*Mamu*) belong to the *MHC-A*, *MHC-B*, *MHC-E*, *MHC-G*, and *MHC-F* types. The characterization of both the MHC-*A* and MHC-*B* genes shows that there is a substantial degree of genetic variability with duplications/deletions of genes and presence of multiple alleles (36–45). Seven *Mamu-A* genes are known. While *Mamu-A1* is present on almost all haplotypes and is the most polymorphic gene in terms of number of alleles (41) (see also IPD database^1^), the other six genes (*Mamu-A2*–*Mamu-A7*) show considerable variability with respect to presence/absence on *Mamu* haplotypes but display only low allelic polymorphism. Thus, in contrast to humans, chimpanzees, and gorillas, the number of *MHC class I* genes on haplotypes is not fixed and contributes significantly to the genetic variability of the rhesus macaque *MHC* (37). Up to six *Mamu-A* genes per haplotype have been observed (43). Assignment of new sequences to one of the seven types of *Mamu-A* genes is rather straightforward and is usually accomplished by phylogenetic tree analysis (39). Similar to *Mamu-A*, the *Mamu-B* genes also show different region configurations and some degree of allelic polymorphism (38, 42–45). So far, only a single *MHC* haplotype has been completely sequenced and contains 19 *Mamu-B* genes of which at least five appear non-functional (36). Studies of segregating sequences in offspring has identified up to 12 transcribed *Mamu-B* genes per haplotype (38, 45). The transcription strength of both *Mamu-A* and *Mamu-B* genes differs, leading to the assignment of genes either as a “major” or “minor” (37, 39). Notably, *Mamu-A1* is a major and the other *Mamu-A* genes are minors. The majority of haplotypes contains only 1–3 *Mamu-B* majors, whereas the number of “minor” *Mamu-B* genes is variable and ranges from 1 to 10 (43). Thus, although the rhesus macaque genome contains a substantial number of *MHC-A* and *MHC-B* type of genes, there appear to be only up to 4 or 5 genes with substantial mRNA production and, most probably, high protein expression on the cell surface.

*Mamu-E* and *Mamu-F* are single copy genes and clear orthologs of the human *HLA-E* and *HLA-F*, respectively, with *Mamu-E* being more polymorphic than *HLA-E* (see IPD database^2^). All *Mamu-G* genes are pseudogenes and the so-called *Mamu-AG* genes, which are a result of a combination of characteristics of both the *Mamu-A* and *Mamu-G* genes, have overtaken their individual function (46, 47). Four potentially functional *Mamu-AG* genes have been identified in the sequenced *Mamu* haplotype (36).

Early studies identified the *MHC class I* allotype Mamu-A1*001 (previous designations *Mamu-A1* or Mamu-A*01) to induce and serve as targets of SIV gag-specific CD8 T cell responses (48–50). Mamu-A1*001-positive rhesus macaques experimentally infected with either SIV_mac_239 or SIV_mac_251 exhibit a delayed progression to AIDS, longer survival times, and lower viral loads at set point as compared to Mamu-A1*001-negative animals (51–54) (Table 1). Similarly, Mamu-A1*002 and A1*011 were found significantly enriched in a cohort of elite controller rhesus macaques infected with SIV_mac_239 (55). By contrast, Mamu-A1*004 (56) and A1*004-positive haplotypes (57) were identified as negatively influencing SIV infection and subsequent progression to disease [odds ratio 11.0 (56)]. Mamu-B*008, B*017, and B*029 have also been identified as beneficial (54, 55, 58) (Table 1). Of note, B*017 and B*029 are frequently observed together in individual rhesus macaques (43, 54, 56, 57, 59) and both genes have been observed as well together with Mamu-A1*001 (54). Thus, the individual contribution of B*017 and B*029 to SIV resistance is difficult to distinguish. Actually, rhesus macaques with identical Mamu-B*017 haplotypes (which probably carry B*029 as well) display substantial variations in chronic-phasic viremia, making it unlikely to predict disease outcome based on the presence of Mamu-B*017 (60). This indicates that a set of combinations of individual *Mamu-A1* alleles and *Mamu-B* region configurations instead of single gene analyses might be more powerful in SIV disease association studies. However, previous association studies had to rely on complex and labor-intensive (Sanger) sequence-based typing of *MHC class I* genes. As a result of this limitation, only a select number of genes were typed in the cohorts of infected and control animals. This limitation has recently been overcome by the advent of massive parallel sequencing, which enables comprehensive *MHC class I* genotyping (42, 56, 61, 62). This technological progress avoids the need to restrict genotyping to a few loci/alleles and, therefore, allows the inclusion of new and previously ignored rhesus macaque *MHC class I* alleles in studies of association analysis.

**Table 1:** **Associations of *MHC class I* and *KIR* genes with experimental SIV infection (SIVmac239, SIVmac251) in rhesus macaques**.

Gene (previous designations)	Effect in SIV infection	Reference
**MHC class I**		
Mamu-A1*001 (A*01)	Lower viral load at set point; fewer loss of CD4+ T cells; longer survival	(51–53, 57)
Mamu-A1*002 (A*02)	More frequently found in elite controllers; longer survival	(55, 57, 58)
Mamu-A1*004 (A*04)	Higher viral load at set point	(56)
Mamu-A3*1303 (A*13)	Longer survival	(53, 57)
Mamu-A1*011 (A*11)	More frequently found in elite controllers; lower viremia in chronic phase of infection	(55, 58)
Mamu-B*001 (B*01)	Not found in elite controllers	(58)
Mamu-B*008 (B*08)	More frequently found in elite controllers; lower viremia in chronic phase of infection	(55)
Mamu-B*017^a^(B*17)	More frequently found in elite controllers; lower viremia in chronic phase of infection	(55, 58)
Mamu-B*029^a^(B*29)	More frequently found in elite controllers; lower viremia in chronic phase of infection	(55, 58)
Mamu-B*047:01 (NB5)	Lower viral load at set point	(53, 57)
Mamu-B*069:01 (NB2)	Lower viral load at set point	(53)
***KIR***		
*KIR3DS* gene copy number variation	Enhanced copies of *KIR3DS*: lower peak viral load but only in animals that are Mamu-A1*001-negative and carry a protective *TRIM5* allele	(63)
*KIR2DL4* gene copy number variation	Enhanced copies of *KIR2DL4*: decreased loss of CD4+ T cells and increased IFN-g production of CD56−CD16− population of NK cells	(64)
*KIR3DL02*	More frequently found in LVL^b^ animals; longer survival; higher numbers of blood NK cells; lower frequency of proliferating blood NK cells, higher percentage of degranulating NK cells	(56)
*KIR3DL05*	More frequently found in HVL^b^ animals; more transcripts found in HVL compared to LVL animals	(56, 65, 66)
*KIR3DL05-KIR3DS05-KIR3DL10*^c^	More frequently found in HVL animals; fewer numbers of blood NK cells; lower percentage of degranulating NK cells	(56)
*KIR3DS01*	Longer survival; higher numbers of blood NK cells	(56)
*KIR3DS02*	More frequently found in HVL animals; shorter survival	(56)
*KIR3DSW08*	More frequently found in LVL animals; longer survival; higher numbers of blood NK cells; lower frequency of proliferating blood NK cells	(56)
***KIR*** ****+**** ***MHC***		
*(KIR3DL05/KIR3DS05/KIR3DL10)* + Mamu-A1*001	Longer survival; lower viral load in gut tissue	(56)
*(KIR3DL05/KIR3DS05/KIR3DL10)* + Mamu-B*012	Shorter survival; higher viral load in gut tissue	(56)

^a^Mamu-B*017 and Mamu-B*029 are closely linked and usually co-occur on haplotypes (43, 54, 56, 57, 59). Therefore, their individual effect in SIV infection is hard to distinguish.

*^b^HVL (high viral load), LVL (low viral load): animals were grouped according to viral load at set point, disease course, CD4+ T cell decline, and other clinical parameters (56)*.

*^c^*3DL05–3DS05–3DL10* are correlated in rhesus macaques (56)*.

### *KIR* Gene Polymorphisms and the Outcome of Experimental SIV Infection in Rhesus Macaques

The rhesus macaque *KIR* region has been completely sequenced using two overlapping BAC clones (67). This sequenced *KIR* haplotype contains five *KIR* genes: *1D, 2DL4, 3DL01, 3DL10*, and *3DL20*. The open reading frame of the *KIR1D* gene mRNA contains exons for the D1 domain, a truncated D2 with premature termination, stem, trans-membrane, and cytoplasmic parts, respectively. However, evidence to date is lacking as to whether this transcribed gene is expressed at the protein level as well. The rhesus macaque *KIR2DL4* gene is orthologous to *KIR2DL4* of hominoids (humans, great, and small apes) and belongs to the *KIR* lineage I. The two rhesus macaque *KIR3D* genes are not particularly related to any of the human or the great ape *KIR3D* genes but are also assigned to the *KIR* lineage II. Phylogenetic tree analysis indicates that there is a mixed relationship between the rhesus macaque *KIR3DL20* with *KIR* genes of human lineage I (*2DL4, 2DL5*) and lineage V (*3DL3*) genes. Exon 3 (D0 domain) of *KIR3DL20* shows closest similarity to human *KIR2DL5*, while the exons 4 and 5 (D1, D2 domain) of *KIR3DL20* are more closely related to human *KIR3DL3*. The *KIR3DL20* exons encoding the stem, trans-membrane, and cytoplasmic regions are more closely related to macaque *KIR* genes (67). Support for a shared phylogenetic origin of the human *KIR3DL3* and rhesus macaque *KIR3DL20* is based on the presence of an identical position at the centromeric end in the *KIR* region of both species (67).

Interestingly, the sequenced haplotype does not appear to encode for any known activating KIR. Further analyses of additional rhesus macaques indicated the presence of activating *KIR* genes, documented the fact that the lineage II *KIR* genes have substantially expanded, and demonstrated differential *KIR* gene content between individuals (56, 62, 65, 67–72). Besides *KIR1D, KIR2DL4*, and *KIR3DL20*, 10 inhibitory *KIR* genes (*3DL01, 3DL02, 3DLW03, 3DL04, 3DL05, 3DL06, 3DL07, 3DL08, 3DL10, and 3DL11*) and 9 activating *KIR* genes (*3DS01, 3DS02, 3DS03, 3DS04, 3DS05, 3DS06, 3DS07, 3DS08, and 3DS09*) were identified. In addition to differential gene content, there is also significant allelic polymorphism of the various *KIR* genes (62, 65, 70, 72). The frequencies of the individual rhesus macaque *KIR* genes within and between populations differ considerably. For example, *KIR3DL01* is present in most animals [84–98% (56, 62, 71)], whereas *KIR3DL04, KIR3DL06*, *KIR3DS01*, *KIR3DS04*, *KIR3DS06*, and *KIR3DS07* are rather rare or even absent in the studied populations (56, 62, 71). As already pointed out, rhesus macaques from different geographic origins differ genetically (31). In accord with this, there are striking differences in the presence of *KIR3DL04, 3DL06, 3DS05*, and *3DS07* genes between Burmese, Chinese, and Indian rhesus macaques (Table 2). Additional differences in the frequencies of other *KIR* genes inherited by macaques of Burmese, Chinese, and Indian origin have been described (71). Such data are indeed useful and important information when analyzing cohorts of animals with known (but also unknown or mixed) geographic origin for studies of genetic association with SIV disease outcome.

**Table 2:** **Prominent differences of *KIR* genes in rhesus macaques from different geographic origin**.

	Geographic origin		
KIR	Burmese (%) (71)	Chinese (%) (71)	Indian (%) (56, 71)
3DL04	0	0	2–10
3DL06	43	94	0–4
3DS05	28	0	50–56
3DS07	31	39	0–9

Specific interactions between MHC class I proteins and a couple of KIR proteins have been described. Thus, the rhesus macaque KIR3D molecules bind to Mamu-A and Mamu-B proteins (15–17). The specificity of the interactions between the lineage II KIR proteins and the corresponding MHC-A and MHC-B ligands is conserved in evolution and has been traced to the divergence of the lineages leading to macaques and humans, i.e., about 31.5 million years ago (73). Similar to human KIR, the binding of rhesus macaque KIR proteins to their cognate ligand is influenced by the peptide content of the ligand (15). Of interest in the context of *KIR* and *MHC class I* polymorphism and SIV infection, Colantonio et al. (15) showed that allotypes of KIR3DL05 differ in their specificity for SIV peptide-loaded Mamu-A1*002 tetramers. Such differences likely contribute to variations in disease susceptibility and the further elucidation of ligand/peptide specificity of the rhesus macaque KIR/MHC ligand system is central to an understanding of the mechanisms underlying the epidemiological disease association data.

The first studies to show an association between *KIR* genes and SIV-induced disease progression identified *KIR3DL05* to be more frequent in animals that rapidly progressed to AIDS (65, 66). However, *KIR3DL05* was later found to be part of a group of closely linked *KIR* genes (*KIR3DL05–KIR3DS05–KIR3DL10*) that were identified in a correlation analysis (56), which makes it difficult to clearly identify one member of this cluster as the responsible gene. Nevertheless, at least one *KIR* gene of this cluster is thought to be responsible for the observed association with various parameters such as high viral load, decreased number of blood NK cells and the lower frequencies of degranulating NK cells (Table 1).

Follow-up studies identified the protective role of *KIR3DL02* and *KIR3DSW08* in SIV infection (56). Thus, animals carrying either one of these two genes (a) slowly progressed to disease or became elite controllers (odds ratio 0.09 and OR = 0.1, respectively), (b) showed longer survival times (hazard ratio 0.24 and 0.29, respectively), (c) exhibited higher number of blood NK cells, and (d) lower numbers of proliferating NK cells (Table 1). Animals having the *KIR3DL02* gene were additionally characterized by a higher conservation of the lytic potential of NK cells. A further beneficial gene might be ascribed to the *KIR3DS01* gene, but the frequency of this gene in the studied cohort was too low to provide robust association data. Future studies are needed that include sufficient number of *KIR3DS01*-positive animals to clarify and establish this association.

Besides gene association studies, several studies have examined copy number variation (CNV) of *KIR* genes to investigate their association with SIV infection outcome (63, 64). It was shown that enhanced copies of *KIR2DL4* is associated with decreased loss of CD4+ T cells and an increase in IFN-gamma production by the CD56−CD16− population of NK cells during the acute phase of SIV infection (64). However, these studies did not investigate whether the CD56−CD16− population indeed expressed the KIR2DL4 protein. It is also conceivable that the observed CNV of *KIR2DL4* is associated with certain *KIR* haplotypes and genes other than *KIR2DL4* could potentially contribute to the associated phenotype. Consistent with this assumption, Hermes et al. (74) found that the CD56−CD16− NK cell subset expressed readily detectable levels of the KIR3D proteins. A further study by Hellmann et al. (63) indicated that higher copy numbers of activating *KIR* genes is associated with lower peak viral load upon SIV infection but only in animals that are Mamu-A1*001-negative and carry a protective *TRIM5* allele.

### Combinations of *KIR* and *MHC Class I* Gene Polymorphisms and the Outcome of Experimental SIV Infection in Rhesus Macaques

There had been no information about epistasis of *KIR* and *MHC class I* genes in rhesus macaques compared to respective information in the human *KIR/HLA* system and HIV-1 infection until a recent study by Albrecht et al. (56) who used 52 SIV-infected rhesus macaques of Indian origin. While not all *KIR* genes could be included in the epistasis analysis due to limited numbers of animals carrying these genes such as *KIR3DL02* or *KIR3DSW08*, the most consistent results were obtained for the linked *KIR3DL05–KIR3DS05–KIR3DL10* genes. The odds ratio of rapid progression to AIDS and presence of *KIR3DS05* or *KIR3DL10* [odds ratio 21.6 (56)] is nullified in animals carrying in addition Mamu-A1*001 [OR = 0.035 for *KIR3DS05*; OR0.036 for *KIR3DL10* (56)]. It appears rather unlikely that the effect is attributable to only Mamu-A1*001, as the risk estimate (i.e., rapid progression to AIDS) for Mamu-A1*001 in animals negative for either *KIR3DS05* or *KIR3DL10* was OR = 1.5 and a considerable number of Mamu-A1*001-positive animals rapidly progressed to disease. Therefore, the authors speculated that the known protective effect of Mamu-A1*001 is more a consequence from epistasis with *KIR* genes than from Mamu-A1*001 alone (56). In addition, there was a potential epistasis of both *KIR3DS05* and *KIR3DL10* with Mamu-B*012 [OR = 25.0, *p* = 0.036 (56)]. Support for the epistases on SIV outcome was obtained by studying also viral copy numbers in gut tissue and the survival time: the simultaneous presence of either *KIR3DS05* and Mamu-B*012 or *KIR3DL10* and Mamu-B*012 is associated with significantly higher viral copy numbers in gut tissue and shorter survival time (hazard ratio 11.1).

Epistasis of *KIR* and *MHC class I* genes and outcome of immunodeficiency virus infection were rather hard to interpret, because interactions between the KIR and corresponding MHC molecules could not be demonstrated (KIR3DS1 and HLA-Bw4-80I) (75). In addition, functional interactions were not performed (KIR3DS05 or KIR3DL10 with Mamu-B*012) (56). However, O’Connor et al. (25) recently demonstrated binding of HLA-B*57 (Bw4-80I positive) tetramers loaded with HIV-1 peptides Pol_839–847_ and Nef_82–90_ to KIR3DS1. This finding is consistent with the idea of activating KIR’s having much lower affinity to self-peptides than pathogen-derived peptides presented on cognate MHC class I proteins. It is generally thought that presentation of peptides influences the relative affinity to both inhibitory and stimulatory KIR’s and, thereby, on the activation of NK cells (76). Higher affinity of inhibitory KIR’s and lower affinity of stimulatory KIR’s to self-peptides would secure tolerance of the NK cell, while presentation of viral peptides would then lead to loss of inhibitory KIR binding (“missing self”) and involvement of activating KIR’s (76–80). It has been speculated that viruses try to evade this NK cell activation by selection of escape mutations enabling stronger binding to inhibitory receptors (76–80). However, it is rather difficult to attribute such escape mutations to only the KIR system and NK cell recognition and not to T cell receptor and CD8+ T cell recognition as both lymphocyte populations use MHC class I proteins as ligands. Nevertheless, from a virus point of view, mutations of MHC class I-presented peptides could kill two birds with one stone and potentially lead to immune escape of both NK and CD8+ T cell responses.

## Conclusion

Products of a number of host genes such as *MHC class I* and *KIR* play crucial roles in the defense of viruses as they are directly involved in the recognition of infected cells and in the restriction of viruses. The enormous genetic variability of both *KIR* and *MHC class I* genes in rhesus macaques impacts the relative susceptibility to SIV infection. The genetic complexity of such host genes, however, formed a major obstacle in the past for molecular typing and comprehensive genetic association analyses. With the advent of next-generation sequencing methods, such limitations are now overcome. Both detrimental and beneficial *KIR* and *MHC class I* genes and alleles as well as combinations of both have already been identified. Further studies will show whether the identified associations can be confirmed in other cohorts and whether further risk or beneficial genes/alleles can be identified. In addition, the KIR/MHC class I ligand system needs further detailed characterization at the protein level, which should allow for a better understanding of the observed genetic associations. Such knowledge would also further strengthen the rhesus macaque SIV infection model of AIDS.

## Conflict of Interest Statement

The authors declare that the research was conducted in the absence of any commercial or financial relationships that could be construed as a potential conflict of interest.

## Footnotes

^1^http://www.ebi.ac.uk/ipd/mhc/nhp/align.html

^2^http://www.ebi.ac.uk/ipd/mhc/
